# Dynamic mechanical loading facilitated chondrogenic differentiation of rabbit BMSCs in collagen scaffolds

**DOI:** 10.1093/rb/rbz005

**Published:** 2019-02-04

**Authors:** Wanxu Cao, Weimin Lin, Hanxu Cai, Yafang Chen, Yi Man, Jie Liang, Qiguang Wang, Yong Sun, Yujiang Fan, Xingdong Zhang

**Affiliations:** 1 National Engineering Research Center for Biomaterials, Sichuan University, 29 Wangjiang Road, Chengdu, China; 2 State Key Laboratory of Oral Diseases, Department of Oral Implantology, West China Hospital of Stomatology, Sichuan University, Chengdu, China; 3 Sichuan Testing Center for Biomaterials and Medical Devices, Sichuan University, 29 Wangjiang Road, Chengdu, China

**Keywords:** bone marrow mesenchymal stem cells, collagen I, dynamic mechanical loading, cartilage tissue engineering

## Abstract

Mechanical signals have been played close attention to regulate chondrogenic differentiation of bone marrow mesenchymal stem cells (BMSCs). In this study, dynamic mechanical loading simulation with natural frequencies and intensities were applied to the 3D cultured BMSCs–collagen scaffold constructs. We investigated the effects of dynamic mechanical loading on cell adhesion, uniform distribution, proliferation, secretion of extracellular matrix (ECM) and chondrogenic differentiation of BMSCs–collagen scaffold constructs. The results indicated that dynamic mechanical loading facilitated the BMSCs adhesion, uniform distribution, proliferation and secretion of ECM with a slight contraction, which significantly improved the mechanical strength of the BMSCs–collagen scaffold constructs for better mimicking the structure and function of a native cartilage. Gene expression results indicated that dynamic mechanical loading contributed to the chondrogenic differentiation of BMSCs with higher levels of AGG, COL2A1 and SOX9 genes, and prevented of hypertrophic process with lower levels of COL10A1, and reduced the possibility of fibrocartilage formation due to down-regulated COL1A2. In conclusion, this study emphasized the important role of dynamic mechanical loading on promoting BMSCs chondrogenic differentiation and maintaining the cartilage phenotype for *in vitro* reconstruction of tissue-engineered cartilage, which provided an attractive prospect and a feasibility strategy for cartilage repair.

## Introduction 

Cartilage plays an essential role of reducing friction, resisting contact wear and cushioning pressure. During joint motion, articular cartilage is in a dynamic and complicated stress environment, including pressure, tension and shear force. This mechanical microenvironment endows the cartilage tissue a series of physical phenomena, such as static pressure gradient, shear stress, deformation of cells and extracellular matrix [1–4]. The articular cartilage is composed of dense collagen fibers, which is an isotropic, heterogeneous and viscoelastic connective tissue, but there are no nerves and blood vessels in articular cartilage. Nutrients could only be obtained from joint fluid, so it is difficult for articular cartilage to self-repair once damaged. To overcome this problem, tissue-engineered cartilage based on stem cells and biomaterials can accelerate the repair of cartilage defect areas, providing the possibility of achieving cartilage regeneration. In clinical application, the mechanical properties of articular cartilage grafts, such as compressive resistance and elasticity modulus, are critical in the process of cartilage remodeling [3, 5, 6]. Regretfully, the artificial cartilage obtained by cartilage tissue engineering can only mimic natural cartilage in morphology and biochemical components, yet its mechanical properties are still quite different from natural cartilage [7, 8].

Bone marrow mesenchymal stem cells (BMSCs) have been considered as ideal seeding cells for cartilage tissue engineering [9]. It has been demonstrated that stem cells are more sensitive to mechanical stimulation than somatic cells, and biomechanical signals are crucial for regulating the phenotype differentiation of stem cells [10–13]. However, conventional tissue-engineering strategies are often lack of mechanical stimulation during cell culture [14–16]. In recent years, mechanical stimulation has been applied *in vitro* culture to promote chondrogenic differentiation and cartilage matrix formation [17–20]. Many studies have demonstrated that stem cells encapsulated in scaffold with dynamic loading stimulation were more analogous to natural tissue [21–24]. Li *et al.* reported that tissue-engineered cartilage constructs cultured in bioreactor could produce more total collagen and sGAG, resulting in greater gain in net tissue weight, as well as expressing cartilage-associated genes, including collagen types II and IX, cartilage oligomeric matrix protein, and aggrecan [23]. Valonen *et al.* used three-dimensionally woven poly(ε-caprolactone) scaffolds combined with adult human mesenchymal stem cells to engineer mechanically functional cartilage constructs *in vitro*. The result indicated that MSC-scaffold constructs by bioreactor-cultured could be endowed with higher collagen content and more homogenous matrix than static controls [24]. Therefore, it has been widely seen that, under mechanical stimulation, more extracellular matrix could be produced by the encapsulated cells, consequently enhancing the mechanical properties of tissue-engineered cartilage to meet the requirements of clinical cartilage repair [25–27].

Scaffold materials can conduce to the adhesion, proliferation and matrix secretion of seeded cells, which is also considered to be crucial ingredient in reconstructing tissue-engineering cartilage. Collagen is widely present in extracellular matrix (ECM), owning plentiful advantages over other scaffold materials in terms of low antigenicity, good biocompatibility, more cell recognition sites, which facilitates cell adhesion and proliferation. Gao *et al.* [28] fabricated excellent cell carrier based on collagen type I and activated chondroitin sulfate by physical and chemical crosslinking without the addition of any catalysts. Furthermore, Zhang *et al.* [29] designed a biocompatible bone repair material prepared from crosslinked porous constructs of collagen and hydroxyapatite, the *in vitro* and *in vivo* experiments results demonstrated the novel materials presented high biocompatibility and effectively induced osteanagenesis ability. Therefore, collagen-based biomaterials are widely applied in cells encapsulation and tissue engineering.

In previous studies, we have carried out a series of studies on the implantation of BMSCs–collagen scaffold constructs for *in vitro* reconstruction of tissue-engineered cartilage [30–32]. In this study, it was expected that the combination of dynamic mechanical loading and BMSCs–collagen scaffold constructs might be promote BMSCs adhesion, uniform distribution, proliferation, secretion of ECM and chondrogenic differentiation, and further enhance the mechanical properties of tissue-engineered cartilage. The cell morphology and mechanical properties were investigated by CLSM and universal mechanical tester. The total GAG production, DNA content and the value of GAG/DNA were tested to determine cell proliferation and ECM secretion. The relevant genes expression including type I, II, X collagen (COL1A2, COL2A1, COL10A1), aggrecan (AGG) and SOX9 was detected to examine chondrogenic differentiation. Matrix secretion and cell morphology and proliferation were further verified by the histological staining.

## Materials and methods

### MSCs isolation and culture

BMSCs were isolated from three neonatal New Zealand rabbits (Breeding Farm for Sichuan Provincial Experimental Animal Special Committee) under sterile conditions as described previously [31]. In brief, the bone marrow was flushed out into culture dishes with culture medium of alpha-modified Eagle’s medium (a-MEM, Hyclone) containing 10% fetal bovine serum (FBS, Gibco) and 100 U/ml penicillin/streptomycin (Hyclone), before cultured in a humidified atmosphere with 5% CO_2_ at 37°C. Non-adherent cells were discarded by changing the culture medium after 24 h. After 70–80% confluence, the cells were passaged and sub-cultured. BMSCs of forth passage were used in the following experiments.

### BMSCs encapsulation

The fabrication of collagen hydrogel encapsulated BMSCs was optimized as our previous work [33]. Collagen solution (extracted from calf skin by dissolution in pepsin/acetic acid, purification by sodium chloride fractionation and final dissolution in 0.5 M acetic acid at 16 mg/ml) was neutralized by 1 M NaOH solution in ice bath and mixed with BMSCs suspension of appropriate amount reaching a final collagen concentration of 6 mg/ml and cell density of 5 × 10^6^ cells/ml. The mixture was then infused into a cylindrical mold (10 mm ϕ, 10 mm high), and the system was transferred in to an incubator at 37°C and kept for 30 min to gel.

### Mechanical loading and culture

Compressive loading was applied using TA ElectroForce BioDynamic 5100 (USA). Briefly, loading was carried out in unconfined compression *via* impermeable stainless steel plates. In order to simulate the real motion state of the organism, the loading protocol consisted of a 10% peak compressive sinusoidal strain at 1 Hz frequency, for 2 h/day. Dynamic compressive loading process was carried out in a humidified incubator at 37°C and 5% CO_2_. The static culture groups were cultured in six-well plates as control. In order to avoid the chondrogenic differentiation medium to interfere with the simple mechanical stimulation, all groups were cultured with α-MEM containing 10% FBS and antibiotics (penicillin 100 U/ml, streptomycin 100 μg/ml), and the medium is changed every 2 days.

### Morphology and mechanical testing of samples

The gross view of each sample was investigated during the culture period at Days 1, 7, 14 and 21, using a digital camera and the sizes were measured using a steel ruler. The elastic modulus of samples was assessed from compression tests using a universal mechanical tester (SANS, China) after culturing for 1, 4, 7, 14 and 21 days, respectively. The samples were compressed to 10% strain with a deformation rate of 1 mm/min. Then the elastic modulus was calculated as the slope of the linear stress strain curve between 5 and 10% strain.

### Cell viability and morphology

After culturing for 1, 4, 7, 14 and 21 days, the samples were harvested and stained by 1 μg/ml fluorescein diacetate (FDA, Topbio Science, China) and 1 μg/ml propidium iodide (PI, Topbio Science, China) for 5 min, and then the fluorescence was visualized by a confocal laser scanning microscope (CLSM, TCSSP 5, Leica, Germany). The live cells are dyed as green by reacting with FDA, while dead cells are dyed as red by PI. The cells’ circularity was analyzed by ImageJ software (1.51j8, National Institutes of Health) based on CLSM pictures.

### Biochemical analysis

Samples were collected and lyophilized at Days 4, 7, 14 and 21. The lyophilized samples were digested by using Papain digestion solution (0.1 mg/ml) at 60°C for 12 h. The supernatants were collected after centrifugation, and the DNA and GAG concentrations were analyzed using a DNA quantitation Kit (Sigma-Aldrich, DNAQK) and a Blyscan™ GAG assay kit (B100, Biocolor).

### Gene expression analysis

The RNA of samples was extracted using a RNeasy Mini Kit (Qiagen) and the concentration was measured in a spectrophotometer (ND1000, Nanodrop Technologies). The extracted RNA was used in the reverse transcription-polymerase chain reaction (RT-PCR), in which an iScript™ cDNA Synthesis Kit (BIO-RAD) was used to reverse transcribe (RT) the extracted RNA to cDNA, and then the SsoFast™ EvaGreen^®^ Supermix (BIO-RAD) was used to conduct the polymerase chain reaction (PCR) on a CFX96 Touch™ Real-Time PCR Detection System (BIO-RAD). The cartilage related genes, including type I, II, X collagen (COL1A2, COL2A1, COL10A1), aggrecan (AGG) and SOX9, where analyzed by normalizing to the housekeeping gene glyceraldehyde-3-phosphate dehydrogenase (GAPDH). Sequences of the primers and probes used are listed in Table 1.

**Table 1:** Primers utilized for qRT-PCR amplification

Target	Forward primer	Reverse primer
GAPDH	5′-TCGGAGTGAACGGATTTGGC-3′	5′-TTCCCGTTCTCAGCTTGAC-3′
COL1A2	5′-GTCGATGGCTGCACGAAAAA-3′	5′-GGGCCAACGTCCACATAGAA-3′
COL2A1	5′-TGATAAGGATGTGTGGAAGCCG-3′	5′-CAGGCAGTCCTTGGTGTCTTC-3′
COL10A1	5′-TCCCAGAACCCAGAATCCATC-3′	5′-GGTTGTGGGCCTTTTATGCC-3′
AGG	5′-GGCCACTGTTACCGTCACTT-3′	5′-GTCCTGAGCGTTGTTGTTGAC-3′
SOX9	5′-TCTGGAGACTGCTGAACGAG-3′	5′-CTGCCCATTCTTCACCGACTT-3′

### Histological analysis

At Days 7, 14 and 21, samples were collected and fixed with 4% paraformaldehyde at 4°C for 24 h. The samples were then washed with PBS for three times, gradient dehydrated in alcohol (50–95%) and then embedded in paraffin. Histological sections (8 μm) were stained with hematoxylin–eosin (H&E) to investigate cell distribution and proliferation. Toluidine blue (TB) staining was used to reveal cell morphology and the composition of synthesized cartilage matrix.

### Statistical analysis

All quantitative results were obtained from at least triplicate measurements. Statistical significance was determined by an analysis with one-way analysis of variance (ANOVA). Significant differences between groups were confirmed with a under 0.05 *P*-values.

## Results

### Morphology of samples

**Figure 1:**
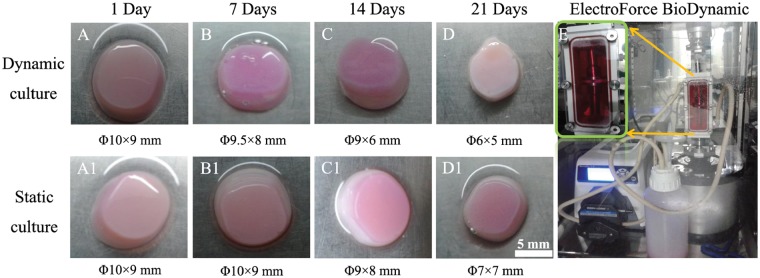
Experimental instrument photograph (E) and the representative images of gross morphology (A–D and A1–D1) of BMSCs–collagen scaffold constructs in dynamic culture group (A–D) and static culture group (A1–D1) at 1, 7, 14 and 21 days; the scale bar: 5 mm

Macroscopic analysis showed that both dynamic culture group and static culture group appeared opaque pink/white in color with smooth surface (Fig. 1). Collagen-based hydrogels presented differences and variations in scaffold dimensional change with extended incubation time between dynamic culture group and static culture group. According to the measurement of hydrogels diameters, slight contractions were observed in both groups for the first week as shown in Fig. 1B and B1. After 2 weeks’ culture, the dynamic culture group (*n* = 3) contracted more significantly from Φ9.5(±0.5) ×8.0(±0.6) mm to Φ6.0(±0.4) ×5.0(±0.3) mm, compared with that of static culture group (*n* = 3) from Φ10.0(±0.3) × 9.0(±0.2) mm to Φ7.0(±0.2) × 7(±0.5) mm. These results indicated that the dynamic loading was likely to promote the contraction of collagen-based hydrogels.

### Mechanical property

**Figure 2:**
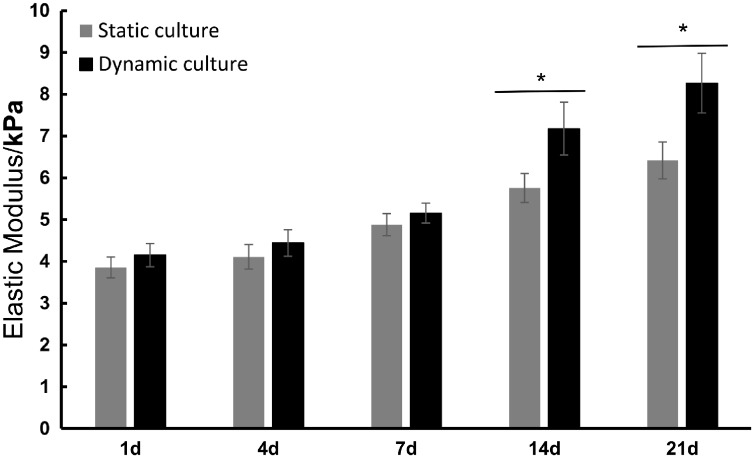
Elastic modulus of BMSCs–collagen scaffold constructs in dynamic culture group and static culture group at 1, 4, 7, 14 and 21 days; data are expressed as the mean SEM (*n* = 3); *P < 0.05

To investigate the change in mechanical strength of the collagen-based hydrogels after dynamic loading or static culture, the hydrogels were removed on Days 1, 4, 7, 14 and 21, respectively, and their Elastic Modulus was tested by a universal mechanical tester. As shown in Fig. 2, in the first week, there was no obvious difference in the mechanical properties between the dynamic culture group and static culture group. However, from Day 14, the elastic modulus of the cell–scaffold complex was significantly increased under mechanical stimulation (*P* < 0.05). These results indicated that the dynamic loading was likely to enhance the mechanical properties of tissue-engineered cartilage.

### Cell viability

**Figure 3:**
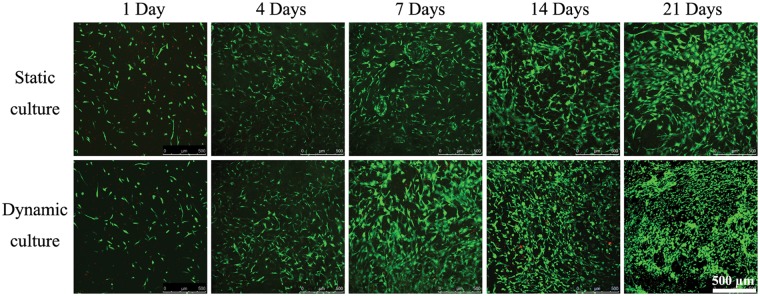
Live/dead staining of BMSCs–collagen scaffold constructs after 1, 4, 7, 14 and 21 days of culture, where the green stain represents the live cells and the red stain represents dead cells; the scale bar: 500

As shown in Fig. 3, live/dead (FDA/PI) staining showed that BMSCs seeded in both groups exhibited excellent viability with few dead cells (in red color), indicating the collagen-based scaffolds were promising to support the seeded BMSCs proliferation. Number of live cells (in green color) significantly increased with time prolongation in both groups. Starting from the second week of culture, the morphology of BMSCs in dynamic culture group exhibited a tendency from typical spindle MSC-like shape to round chondrocyte-like shape. Whereas in static culture group, it appeared that the cells were spindle shaped and became more spread, especially at Day 21. The cellular circularity for cells in dynamic culture group compared with those in static culture group at 1, 4, 7, 14 and 21 days were listed in Table 2.

**Table 2:** The circularity of cells in dynamic culture group and static culture group at 1, 4, 7, 14 and 21 days were presented

Time (days)	Static culture	Dynamic culture
1	0.588 ± 0.269	0.605 ± 0.271
4	0.442 ± 0.182	0.440 ± 0.207
7	0.395 ± 0.184	0.319 ± 0.201
14	0.340 ± 0.185	0.548 ± 0.278
21	0.329 ± 0.196	0.600 ± 0.270

Dynamic mechanical loading facilitated the rabbit BMSCs encapsulated in collagen hydrogel adhesion, uniform distribution, proliferation and secretion of ECM. Dynamic mechanical loading also significantly improved the mechanical strength of the BMSCs–collagen scaffold constructs and prevented of hypertrophic process and reduced the possibility of fibrocartilage formation by gene expression analyzation.

### Biochemistry analysis

**Figure 4:**
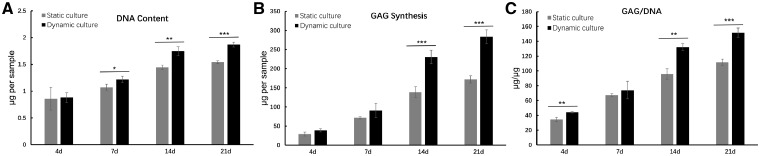
The values of DNA (**A**), GAG (**B**) and GAG/DNA (**C**) in dynamic culture group and static culture group at 4, 7, 14 and 21 days. Data are expressed as the mean SEM (*n* = 3); *P < 0.05, **P < 0.01 and ***P < 0.001

Total GAG production, DNA content and the value of GAG/DNA were presented as shown in Fig. 4. The DNA content results showed an increasing trend in both dynamic culture group and static culture group with time prolonging. However, the DNA content was significantly higher in dynamic culture group than that of static culture group after 7 days culture (*P* < 0.05 in 7 days, *P* < 0.01 in 14 days, *P* < 0.001 in 21 days) (Fig. 4A). Similarly, mean GAG production was quickly accumulated with time increasing in both groups, and significantly higher GAG synthesis was detected in dynamic culture group compared with static culture group after 14 days culture (*P* < 0.001 in 14 and 21 days) (Fig. 4B). GAG/DNA content in dynamic culture group and static culture group indicated the cartilage matrix synthesized by BMSCs (Fig. 4C). The GAG/DNA content improved with increasing time in both groups. From 14 days, GAG/DNA in the dynamic culture group was significantly higher than the static culture group (*P* < 0.01 in 14 days and *P* < 0.001 in 21 days). These data were agreed with the visible results in Fig. 3.

### Chondrogenic gene expression

**Figure 5:**
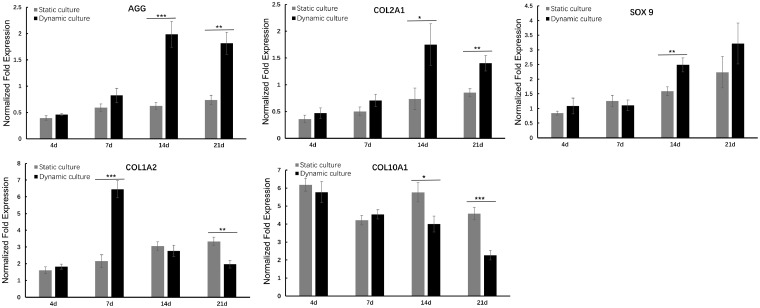
The genes expression of COL1A2, COL2A1, COL10A1, AGG and SOX9 in dynamic culture group and static culture group at 4, 7, 14 and 21 days were presented, respectively. All gene expressions were normalized to housekeeping gene GAPDH; data are expressed as the mean SEM (*n* = 3); *P < 0.05, **P < 0.01 and ***P < 0.001

The relevant genes expression was shown in Fig. 5. Compared with static culture group, the COL2A1 and AGG expression of BMSCs under dynamic loading significantly increased after 14 days, even up to ∼3-fold in the second week (*P* < 0.001 for AGG and *P* < 0.05 for COL2A1), and about double folds in 21 days (*P* < 0.01). The expression of SOX9 in both static culture and dynamic culture group generally showed an upward trend over time. At the first week, SOX9 expression in the two groups was relatively close, and no significant difference was found. And at the second week, SOX9 expression of dynamic culture group was obviously higher than that of static culture group (*P* < 0.01).

The expression of COL1A2 in the static culture group kept slowly increasing in the whole culture cycle. Interestingly, a significant rise was found in the seventh day in dynamic culture group, and subsequently progressive decline appeared, even only 60% of expression level of COL1A2 was detected in 21 days, in compared with that of static culture group. Accordingly, the expression of COL10A1 presented an overall gradually decreased tendency with time extension in dynamic culture group while in static culture group, no obviously decrease was observed. It was noteworthy that the dynamic culture group showed significantly lower gene expression levels than that of static culture group from 14 days (*P* < 0.05 in 14 days and *P* < 0.001 in 21 days).

### Histological analysis

**Figure 6:**
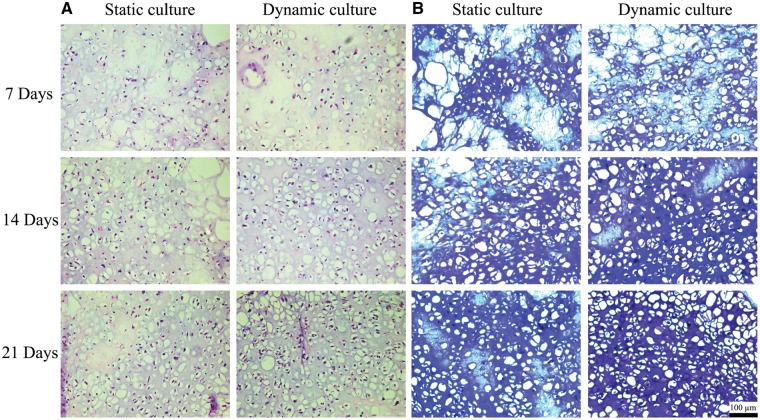
H&E staining (**A**) and TB staining (**B**) of samples in dynamic culture group and static culture group at 7, 14 and 21 days were presented; the scale bar: 100 μm

H&E and TB staining of dynamic culture group and static culture group after 7, 14 and 21 days culture were shown in Fig. 6. The results of H&E staining showed that with the increase of culture time, the number of cells gradually increased in both groups, while the cells distribution in the dynamic culture group was more uniform than that of static culture group. TB could stain the cartilage specific polysaccharides and acidic proteoglycan purple. With the extension of culture time, the staining degree of TB gradually increased in both groups, but the heavy staining of TB was presented in dynamic culture group from 14 days.

## Discussion

In recent years, the rapid development of cartilage tissue engineering has brought hope for repair large-area cartilage damage [1, 3, 5]. Through tissue engineering, the cultured cell–scaffold constructs could be used as a cartilage graft to repair damaged cartilage [5]. Bioreactors can be used to increase the amount of fluid flowing inside and outside the scaffold [4]. Besides increasing cell viability, another important role of bioreactors was to stimulate cells to produce more extracellular matrix (ECM) in a shorter period through corresponding physiological reaction of cells under certain mechanical stimulation. Meanwhile, the uniform distribution of cells could also be guaranteed [6, 21]. Moreover, the increase of extracellular matrix was more conducive to improving the mechanical strength of the tissue-engineered cartilage [34]. In this study, dynamic mechanical loading simulation with natural frequencies and intensities were applied to the 3D cultured BMSCs in a bioreactor.

As shown in Fig. 1, the gradually decreased volume of cell–scaffold complexes could be explained by the enhanced interaction between seeded cells and scaffolds. During the 3D culture, BMSCs adhering to the scaffold would exert contractive tension and secret various enzymes, resulting in the shrinkage of the scaffold [33]. The behavior of contraction would also lead to the increased cell density and the enhanced cell–cell interactions. The dynamic culture group contracted more than static culture group after 21 days culture, implying the increased cell population and cell secretory activity. Figure 2 shows the elastic modulus of the cell–scaffold constructs with or without dynamic loading at different time points. The additional imposed mechanical properties on cell–scaffold constructs in dynamic culture group presented significant mechanical enhancement, which probably further promoted the production of more abundant ECM. To a certain extent, it was more conducive to construct bionic natural cartilage tissue utilizing principles of biological science, regenerative medicine and engineering.

These results of live/dead (PI/FDA) staining (Fig. 3) indicated that more visual cell proliferation in dynamic culture group has been observed than static culture group. The results agreed with previous work that emphasized the importance of dynamic culture on cell proliferation using hMSC-laden hyaluronic acid hydrogels [34]. Moreover, from the 14th day, the cell morphology gradually changed from MSC-like long spindle type towards chondrocyte-like circular type in dynamic culture group, while there was no obvious change of cell morphology in the static culture group, demonstrating that mechanical stimulation could promote the differentiation of BMSCs into chondrocyte-like cells. As shown in Fig. 4, the dynamic culture group revealed higher GAG production, DNA content and the value of GAG/DNA. These data showed that mechanical stimulation was beneficial for cells to secrete more extracellular matrix and promote cell proliferation, which kept consistent with the previous results.

Gene expression of chondrogenic phenotype markers in the dynamic culture and static culture groups was analyzed by qPCR at each time point. In general, all the three chondrogenic markers (AGG, COL2, SOX9 genes) were expressed at higher levels in dynamic culture group compared with static culture group (Fig. 5), suggesting that dynamic loading contributed to the chondrogenic differentiation of BMSCs. COL1A2 is a maker related to cell adhesion, migration and osteogenesis [35]. Interestingly, the COL1A2 gene expression in dynamic culture group was remarkedly high at Day 7 and gradually decreased with time extension. As the dynamic loading facilitate the migration and proliferation of BMSCs at the early stage (Fig. 3), it was not surprised to see the increased COL1A2 expression at the same time point. However, once the cell density reached the critical point, cell started to aggregate and initiated chondrogenic differentiation [36, 37]. Consequently, the cell proliferation marker of COL1A2 decreased after Day 7, and all three chondrogenic markers increased and maintained at a high level at both Days 14 and 21. This result also well agreed with the obviously higher expression of COL2A1 and AGG. Together with the increased GAG/DNA after the second week of culture, a higher rate of chondrogenic differentiation was achieved after BMSCs adhesion and distribution. In MSC-based cell therapy for cartilage repair, it is crucial to maintain chondrocyte-like morphology in the neo-cartilage tissue. One of the major challenges is that the MSC-derived chondrocyte-like cells may undergo hypertrophy and eventually form mineralizing matrix [34, 38, 39]. COL10A1 is considered as a marker of cartilage hypertrophy and endochondral bone formation. Dynamic mechanical stimulation could down-regulate the expression of COL10A1, which might contribute to prevent of hypertrophic process during chondrogenic differentiation, which was consistent with previously reports [34, 40].

In line with the results of live/dead staining, H&E staining (Fig. 6) showed that pressure stimulation could promote the proliferation of BMSCs in 3D culture and homogenize the even distribution of cells in scaffold. TB stained the nucleus in blue and the cartilage specific matrix in purple (Fig. 6), which indicated the deposition of proteoglycan and GAG in the matrix of the sample. With the increase of culture time, purple color gradually reinforced, and the color of dynamic culture group was heavier than that of static culture group, indicating that BMSCs tended to differentiate into chondrocyte-like cells and secrete more ECM under dynamic loading.

## Conclusion

In this study, dynamic mechanical loading simulation with natural frequencies and intensities were applied to the 3D cultured BMSCs–collagen scaffold constructs. Compared with the static culture group, dynamic mechanical loading facilitated the BMSCs adhesion, uniform distribution proliferation and secretion of ECM even though there was a slight contraction, which significantly improving the mechanical strength of the BMSCs–collagen scaffold constructs for better mimicking the structure and function of native cartilage. Gene expression results indicated dynamic mechanical loading contributed to the chondrogenic differentiation of BMSCs with higher levels of AGG, COL2A1 and SOX9, and prevented of hypertrophic process with lower levels of COL10A1, and reduced the possibility of fibrocartilage formation due to lower levels of COL1A2. To summarize, dynamic loading during the culture of BMSCs encapsulated collagen hydrogel provides an attractive prospect and a feasibility strategy for *in vitro* reconstruction of tissue-engineering cartilage.

## Funding

This work was supported by the National Key Research Program of China (2018YFC1105901), Young Elite Scientists Sponsorship Program by CAST (2017QNRC001), the 111 Project (No. B16033), and the Sichuan Science and Technology Program (2018RZ0039).

*Conflict of interest statement*. None declared.
